# Supplementary material for: Comparison of enteric methane yield and diversity of ruminal methanogens in cattle and buffaloes fed on the same diet
Source: PLoS One. 2021 Aug 11;16(8):e0256048. doi: 10.1371/journal.pone.0256048 (PMC8357158; doi:10.1371/journal.pone.0256048)
Supplement: S1 Table — (DOCX) [file pone.0256048.s001.docx]

**S1 Table. Chemical composition of feed**

| **Constituent** | **Napier grass** | **Concentrate** |
| --- | --- | --- |
| Crude protein (g/kg DM) | 63 | 230 |
| Neutral detergent fibre (g/kg DM) | 780 | 325 |
| Acid detergent fibre (g/kg DM) | 410 | 100 |
| Ash (g/kg DM) | 122 | 63 |
| Organic matter (g/kg DM) | 878 | 937 |
