# Supplementary figures and images for: Comparison of enteric methane yield and diversity of ruminal methanogens in cattle and buffaloes fed on the same diet
Source: PLoS One. 2021 Aug 11;16(8):e0256048. doi: 10.1371/journal.pone.0256048 (PMC8357158; doi:10.1371/journal.pone.0256048)

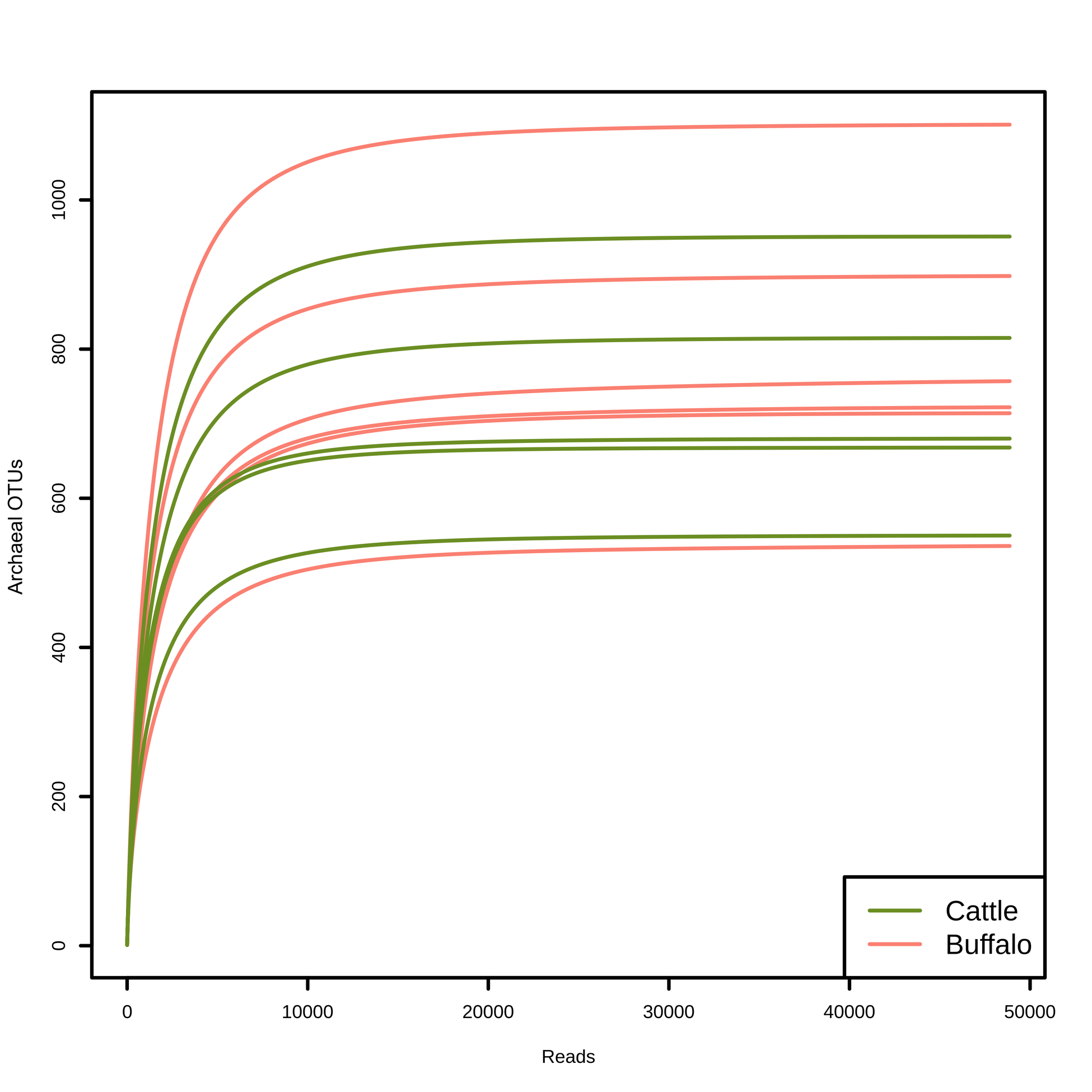

Supplement: S1 Fig — (TIF) [file pone.0256048.s002.tif]
