# Supplementary material for: Comparison of enteric methane yield and diversity of ruminal methanogens in cattle and buffaloes fed on the same diet
Source: PLoS One. 2021 Aug 11;16(8):e0256048. doi: 10.1371/journal.pone.0256048 (PMC8357158; doi:10.1371/journal.pone.0256048)
Supplement: Attachment — Submitted filename: Malik et al PlosOne 27-4-21 with Reviewer commenrs and edits .docx [file pone.0256048.s004.docx]

**Comparison of Enteric Methane Emissions and Diversity of Ruminal Methanogens**

**in Cattle and Buffaloes fed on the Same Diet**

P.K. Malik^1*^, S. Trivedi^1^, A. Mohapatra^1^, A.P. Kolte^2^,

V. Sejian^3^, R. Bhatta^1^ and H. Rahman^4^

^1^ Bioenergetics and Environmental Science Division, National Institute of Animal Nutrition and Physiology, Bangalore, Karnataka, India

^2^ Animal Nutrition Division, National Institute of Animal Nutrition and Physiology, Bangalore, Karnataka, India

^3^ Animal Physiology Division, National Institute of Animal Nutrition and Physiology, Bangalore, Karnataka, India

^4^ International Livestock Research Institute, South Asia, New Delhi, India

* Corresponding author

E-mail: malikndri@gmail.com (PKM)

All the authors contributed equally to this work

**Comparison of Enteric Methane Emissions and Diversity of Ruminal Methanogens**

**in Cattle and Buffaloes fed on the Same Diet**

**Abstract**

An *in vivo* study was conducted to compare the enteric methane emissions and diversity of ruminal methanogens in cattle and buffaloes kept in the same environment and fed on the same diet. Six cattle and six buffaloes were fed on a similar diet comprising Napier (*Pennisetum purpureum*) green grass and concentrate in 70:30. After 90 days of feeding, the daily enteric methane emissions were quantified by using the SF_6_ technique and rumen fluid samples from individual animals were collected for the diversity analysis. The daily enteric methane emissions were significantly greater in cattle as compared to buffaloes; however, methane yields were not different between two species. Methanogens were ranked at different taxonomic levels against the rumen intestinal methanogens database. The archaeal communities in both host species were dominated by the phylum *Euryarchaeota*; however, *Crenarchaeota* represented <1% of the total archaea. Methanogens affiliated with *Methanobacteriales* were most prominent and their distribution did not differ between the two hosts. *Methanomicrobiales* and *Methanomassillicoccales* constituted the second largest group of methanogens in cattle and buffaloes, respectively. *Methanocellales* (*Methanocella arvoryza)* were exclusively detected in the buffaloes. At the species level, *Methanobrevibacter gottschalkii* had the highest abundance (55-57%) in both the host species. The relative abundance of *Methanobrevibacter wolinii* between two hosts varied significantly. *Methanosarcinales*, an acetoclastic methanogens, were significantly higher in cattle than the buffaloes. With the diet used in this study, there was limited influence (<3.5%) of the host on the structure of the ruminal archaeal community at the species level. Therefore, the methane mitigation strategies developed in either of the hosts should be effective in the other. Further studies are warranted to study the conjunctive effect of diet and geographical locations with the host on rumen archaeal community composition.

**Keywords:** Buffalo, Cattle, Methanogens diversity, Rumen archaea, 16s rRNA

**Introduction**

Methane is the second most significant greenhouse gas in the atmosphere [1]. The present atmospheric concentration of methane is about 1830 ppb which is increasing at an average rate of 10 ppb per year [2]. Enteric fermentation with an annual emission of 80-90 Tg (teragram) methane remains one of the largest sources in the agriculture sector. The contribution of cattle and buffaloes to the annual enteric methane emission is estimated to be 72 and 11 Tg, respectively. India has about 13% of the global population of cattle and 53% of the global population of buffaloes [3] and these account for 4.92 and 2.91 Tg of annual global enteric methane emission from the respective species [4]. These two major bovine species are responsible for more than 85% of the enteric methane emission in India. Methanogenesis acts as a major sink for the fermentative H_2_ in the rumen; nevertheless, due to the loss of energy, it is considered wasteful process. As each litre of methane produced in the rumen carries 39.5KJ energy [5]; therefore, 2-12% of the biological energy is just wasted in the form of methane [6].

Despite the limited representation of archaea (3-5%) in the rumen [7,8]; they play a significant role in maintaining the H_2_ pressure low within the desirable limits [9,10]. Unlike bacteria, archaea do not have peptidoglycan in the cell wall [11]. A unique characteristic of methanogens is coenzyme F_420_ [12], which facilitates the redox transformations [13]. Though the rumen methanogens community is not as diverse as bacteria; nevertheless, the contrast substrate requirement for different categories makes this community complex. Hence, understanding the rumen archaeal community is crucial for devising effective methane mitigation strategies.

Host impact on the ruminal microbiota establishment has been reported previously [14–18]. Environmental conditions have also been reported to influence the ruminal archaeal community [19]. In a global study, [20] narrated that *Methanobrevibacter gottschalkii,* *Methanobrevibacter ruminantium* clades, *Methanosphaera* sp. and two *Methanomassiliicoccaceae*-affiliated groups with variable abundance dominate the archaeal community in all animal species globally. Due to the variable physical and chemical characteristics of feed, diet remains a major determinant that shapes the microbial community in the host animal [8]. In contrast, some remarkable differences were reported in the dominance of rumen archaea in the animal host [21–24].

From these studies, it remains uncertain whether methodological variation, host, diets, or geographical conditions led to the difference and similarities in the archaeal community. Host factors are thought to be important in shaping the archaeal community in the rumen. However, there is a dearth of information demonstrating the impact of host species on the rumen archaea with similar feeding and environmental conditions. To disallow any variation in the archaeal community due to the diet and environmental conditions, a study was designed to investigate and compare the archaeal community composition in cattle and buffaloes fed on the same diet under the same environmental/geographical conditions.

**Materials and Methods**

**Animals and feeding**

The experiment was conducted at the Livestock Experimental Station of the institute situated in the Deccan Plateau at an average elevation of 900 m at 12.97°N and 77.56°E. All the procedures involving animals were performed as per the guidelines and regulations of the Institute Animal Ethics Committee (NIANP/IAEC/1/2020/5). An *in vivo* study to compare the enteric methane emission and the composition of the rumen methanogen community was conducted in six adult male cattle (BW 538±23.3 kg) and six adult male buffaloes (BW 284±14.5 kg). To disallow any variation in the diversity due to geographical region, both the cattle and buffaloes were kept in the same environmental conditions during the entire experimental period. The average minimum and maximum temperatures during the experimental period (April-June) were 21.8 and 35.8°C; while average humidity was 57%. The animals were housed in a well-ventilated experimental shed and dewormed orally with fenbendazole (5mg kg^-1^) before the beginning of the experiment. Another important factor that affects the composition of the methanogen community in the rumen is diet variation; hence, in the present study, animals of both species were fed on a similar diet comprising Napier (*Pennisetum purpureum*) green grass and concentrate in the ratio of 70:30. The concentrate mixture was prepared using maize grain (32 parts), Soybean meal (13 parts), groundnut cake (12 parts), wheat bran (40 parts), mineral mixture (2 parts), and salt (1 part). Experimental animals were offered the basal diet daily at 09.00h; while clean drinking water was accessible to the animals throughout the day. The dried and ground feed and concentrate samples were analyzed for crude protein (Nx6.25) and ash content as per [25]. However, the fibre constituents such as neutral detergent fibre (NDF) and acid detergent fibre (ADF) were determined according to [26]. The chemical composition (% dry matter basis) of the green fodder and concentrate is given in S1 Table.

**Methane emission**

After 90 days of feeding, the daily enteric methane emission in cattle and buffaloes was quantified by using sulfur hexafluoride (SF_6_) tracer technique [27,28] for the 7 days. During the methane measurement study, the dry matter intake for the individual animal was recorded by considering the amount of feed offered and refusals. The concentration of methane and sulfur hexafluoride gases in breath and background samples was measured using a gas chromatograph (GC 2010 plus, Shimadzu, Japan) fitted with flame ionization and electron capture detectors, respectively. Before analysis, the samples were diluted with N_2_ gas and thereafter a representative sample was drawn from the canister using a Hamilton airtight syringe and injected into the gas chromatograph for estimation of methane and sulfur hexafluoride. The enteric methane emission was calculated using the following formula [27].

$$\text{QCH}_{\text{4}}\text{ =}\text{QSF}_{\text{6}}\text{×(}\frac{\text{CH}_{\text{4}}}{\text{SF}_{\text{6}}}\text{)}$$

QCH_4_: daily CH_4_ output (g/d); QSF_6_: SF_6_ release rate from permeation tube; CH_4_/SF_6_: mixing ratios of CH_4_ and SF_6_ in collected breath samples.

The data generated were analyzed using IBM SPSS Statistics for Windows, (Version 21.0. Armonk, NY: IBM Corp.) and following the analysis of variance (ANOVA). The difference between the means was compared by Tukey’s method and considered significant at P≤0.05.

**Ruminal Fluid Collection**

At the end of methane measurement trial, the rumen fluid samples were collected from each animal (~45 ml) using a stomach tube. The collected samples were filtered through double layers of muslin cloth and placed on the ice before transporting to the laboratory for further processing. The samples were brought to the laboratory for further processing. Each sample was divided into three equal subsets. The first subset of rumen fluid was processed for the ammonia-N (NH_3_-N) and volatile fatty acid (VFA) estimation; while the second and third subsets were used for the DNA isolation and protozoal enumeration respectively.

**Estimation of VFA and ammonia N**

The volatile fatty acid concentration in the rumen fluid samples was determined as per Filipek and Dvorak [29] using a gas chromatograph (Agilent, 7890A) fitted with a flame ionization detector and capillary column (40 m x 0.18 µm). The concentration of NH_3_-N in the samples was determined following the procedure of [30].

**Protozoal Enumeration**

The enumeration of protozoa in rumen fluid was performed using a Neubauer counting chamber according to the method described previously [31]. The rumen protozoa were categorized into *Entodiniomorphs* or *Holotrich* based on the gross morphology and distribution of cilia over the body surface [32].

**DNA Isolation**

The frozen rumen liquor samples were thawed at room temperature, centrifuged at 1000g for 5min to allow the sedimentation of dissolved micro feed particles, and supernatants were collected. About 2 mL supernatant was taken into an *Eppendorf* tube and centrifuged at 4°C, 13400 rpm for 10 minutes. A thick pellet obtained by the centrifugation was retained while removing the supernatant carefully. Subsequently, 1 mL lysis buffer was added to it and dissolved pellet through gentle pipetting. The mixed content was transferred to a 2 mL sterile screw cap tube contained 0.5 g (0.1 mm) pre-sterilized zirconia beads (Biospec USA). The repeat bead beating plus column method [33] was used for the genomic DNA isolation in the present study. The QIAamp DNA mini kit (Qiagen, Germany) was used as per the manufacturer’s instructions. Quality of the genomic DNA was checked using 0.8% agarose gel electrophoresis; while the DNA concentration was confirmed with Qubit 4.0 (Invitrogen).

**Library Construction and Sequencing**

Genomic DNA samples were processed for the preparation of amplicon libraries and sequencing. Amplicon libraries were prepared using Nextra XT kit (Illumina Inc.). Archaea specific primers *Arch-344F* 5’ACGGGGYGCAGCAGGCGCGA 3’ [34] and *Arch-806R* 5’GGACTACVSGGGTATCTAAT 3’ [35] were used for the amplification. Illumina adapters i5 and i7 were added to the primers for generating the amplicons. Amplicon libraries were purified by AMPureXP beads (Beckman Coulter Life Sciences, USA) and analyzed on 4200 Tape Station system (Agilent Technologies, USA) using D1000 Screen Tape station. About 10-20 pM of each library was loaded on to the MiSeq platform for cluster generation and sequencing.

**Bioinformatics analysis**

Raw amplicon sequence reads generated from Miseq were processed using DADA2 V1.16 [36] in R V4.0.2. Raw reads quality was assessed using function plot Quality Profile followed by dereplication, denoising, and merging of the paired reads. The Truncation parameter was set to 295 and 260 nucleotides for the forward and reverse reads respectively. Chimeras were removed from the filtered reads using function removeBimeraDenovo by implementing the consensus method with minFoldParentOverAbundance. DADA2 compatible reference fasta file for taxonomy assignment was generated using RIM-DB [37] by an in-house python script. Further, taxonomy classification was performed on the reads using assign taxonomy function against the RIM-DB. An annotated taxonomy table from DADA2 was imported into phyloseq package V1.26.1 [38] in R for the further downstream analysis. Low abundance OTUs (operational taxonomic unit) were pruned and samples were rarefied to the lowest read numbers of samples to examine the archaeal diversity measures. The rarefaction curve was plotted using the rarefy function from vegan package [39]. Alpha diversity measure was estimated by the Shannon index and *post hoc* comparison was performed using pairwise Wilcoxon ranksum test. To test the multivariate homogeneity of group dispersion, betadisper function from vegan package was implemented. Further, principal coordinate analysis was performed based on the Bray-Curtis dissimilarity matrix and *post hoc* comparison was done using Permutational Multivariate Analysis of Variance (PERMANOVA) by adonis function. The OTUs abundance at different taxonomic ranks was studied between two host species cattle and buffaloes and the relative abundance plots were generated using ggplot2 [40]. The OTU count data was normalized and differential abundance significance was tested at different taxonomic ranks using Wald parametric test with Benjamin-Hoschberg correction from DESeq2 [41]. Core microbiome analysis was also performed using package microbiome V1.4.1 [42] in R with a minimum prevalence and detection threshold of 50% and 0.01, respectively.

**Data availability statement**

The archaeal metagenome sequencing reads from the experiment are accessible at the NCBI Sequence Read Achieve (SRA; [https://www.ncbi.nlm.nih.gov/subs/sra](https://www.ncbi.nlm.nih.gov/subs/sra/SUB7668476)) accession numbers SAMN16378101- SAMN16378111 under BioProject PRJNA667560. The OTUs abundance and taxonomical assignment data are available in S1 Appendix.

**Results**

**Dry matter intake and methane emission**

*In vivo* study revealed that the enteric methane emission (g/d) was significantly greater (p<0.001) in cattle as compared to the buffaloes. Similarly, a higher (p<0.001) dry matter intake was also recorded in the cattle than the buffaloes (10.5 Vs. 6.86 kg/d). However, when methane was expressed as methane yield so that emissions are expressed per unit of dry matter intake (g/kg DMI) methane yields were similar (Fig 1) between cattle (13.4 g/kg DMI) and buffaloes (13.5 g/kg DMI) fed on similar diet comprising hybrid Napier and concentrate in 70:30.

**Fig 1** Panel A illustrates the variation in enteric methane (g/d & g/kg DMI), while panel B represents the dry matter intake in both cattle (**C**) and buffaloes (**B**)

**Rumen fermentation and protozoal concentration**

This study did not reveal any measurable variation in the concentrations (mM/l) of rumen ammonia (mg/100 ml) and total volatile fatty acids. Similarly, the individual fatty acid concentration except iso-butyrate was also comparable between the two host species (Table 1) fed on a similar diet consisting of Napier grass and concentrate. The numbers (x10^6^/ml) of total protozoa and *Entodiniomorphs* were higher (p<0.05) in the buffaloes. However, the *Holotrichs* numbers (x10^5^/ml) were comparable between the two host species.

**Table 1** Comparative rumen fermentation characteristics profile between two host species

| **Attributes** | **Cattle** | **Buffalo** | **P value** |
| --- | --- | --- | --- |
| Ammonia N (mg/100 ml) | 8.63 | 9.10 | 0.739 |
| TVFA (mM/l) | 93.6 | 57.3 | 0.152 |
| **Individual VFA (molar proportion)** | | | |
| Acetate | 60.7 | 61.4 | 0.639 |
| Propionate | 15.2 | 14.7 | 0.391 |
| Butyrate | 19.2 | 18.9 | 0.826 |
| Iso-butyrate | 0.91^a^ | 1.05^b^ | 0.025 |
| Valerate | 2.52 | 2.75 | 0.241 |
| Isovalerate | 1.41 | 1.08 | 0.243 |
| **Protozoa** | | | |
| Total (x10^6^/ml) | 23.6^a^ | 36.1^b^ | 0.014 |
| *Entodiniomorphs* (x10^6^/ml) | 23.4^a^ | 35.8^b^ | 0.014 |
| *Holotrichs* (x10^5^/ml) | 1.65 | 2.26 | 0.318 |

TVFA- total volatile fatty acid, VFA- volatile fatty acid, the mean values are in columns are presented along with standard error

**Effect of the host on methanogens community**

A total of 2,628,682 archaeal raw reads with an average of 238,971 reads per sample were generated from the study. After quality filtering and chimera removal, a total of 827,901 reads was retained for further analysis (S1 Appendix). Taxonomy classification of reads at ≥97% similarity against the RIM-DB clustered produced a total of 3,924 archaeal OTUs (S1 Appendix). Rarefaction plot obtained from the samples in this study confirmed that the diversity of rumen methanogens in both the hosts was adequately covered (Fig 2).

**Fig 2 Rarefaction plot showing the number of OTUs**

All filtered reads in the present study were affiliated to the archaea. Taxonomic annotation of OTUs revealed that the rumen archaeal community was dominated by the phylum *Euryarchaeota* in both cattle (98%) and buffaloes (97%). In this study, the phylum *Crenarchaeota* representation was only <1%. The relative abundance of rumen methanogens at different taxonomic ranks is presented Figs 3 and 4.

**Fig 3** Rumen archaeal community composition in cattle and buffaloes at (a) the order level and (b) family level. The inner-circle represents the methanogens distribution in cattle; while the outer circle represents the distribution in buffaloes

**Fig 4** Total rumen archaeal methanogen community at genus and species levels in cattle and buffaloes. Each genus is represented by larger bars that are underlaid on the smaller bars representing the abundance of all the species affiliated to the corresponding genus

About 22-24% of the reads irrespective of the host at order and class levels remain unclassified. Methanogens belonging to six orders were identified in the cattle; while there were seven orders in the buffaloes. The Methanogens associated with the *Methanocellales* were exclusively identified in the buffaloes. The methanogens affiliated to the *Methanobacteriales* were dominant in both the host and represented 72-73% of the total rumen archaea (Fig 3a). There was no difference (P= 0.872) in the distribution of *Methanobacteriales* between the cattle and buffaloes. In cattle, *Methanomicrobiales* were the second most abundant methanogens (3.75%); whilst they constituted only 0.85% of the total archaea in buffaloes. In buffaloes, *Methanomassillicoccales* represented the second largest group of methanogens. However, the distribution of either *Methanomicrobiales* (P= 0.245) or *Methanomassillicoccales* (P= 0.872) did not differ between the two host species. Though the *Methanosarcinales* were distributed at a lower frequency in both the cattle and buffaloes; nevertheless, their abundance was significantly higher (P= 1e^-6^) in the cattle as compared to buffaloes. Morphologically and physiologically distinguished archaea belong to the order *Sulfolobales* (*Crenarchaeota)* were also identified in both host species.

Methanogens belonging to a total of nine families were identified in this study (Fig 3b). All the orders were represented by one family; however, the orders *Methanomicrobiales* and *Methanosarcinales* in both host species were characterized by the two families of each. Most of the orders had only single taxa classified at the family level and thus precisely represented the same relative abundance at both hierarchy levels (order and family). *Methanomicrobiaceae* and *Methanocorpusculaceae* were affiliated to the *Methanomicrobiales*; while *Methanosarcinaceae* and *Methanosaetaceae* were classified within the order *Methanosarcinales*. At the family level, the distribution of *Methanosarcinaceae* methanogens only was significantly higher (P=1e^-6^) in cattle than in buffaloes (Fig 3b; S1 Appendix).

In the present study, 14 genera of the methanogens were identified in cattle; while archaea associated with 12 genera were identified in buffaloes. The *Methanobrevibacter* irrespective of the host species was the most prominent genus and represented 66-68% of the total archaea in the rumen. However, their distribution between cattle and buffaloes was comparable. Methanogens affiliated to the *Methanobacterium* and *Methanomicrobium* were the second and third largest genus in the cattle and aggregately constituted 7.3% of the archaea. On the other hand, *Methanosphaera* and *Methanobacterium* were the second and third largest genus of methanogens in buffaloes, respectively, and aggregately constituted about 5% of the rumen archaea. The *Methanosaeta* and group 8 methanogens were distributed at a low frequency and exclusively detected in the rumen of cattle. On the other hand, *Methanocella* were detected in the buffaloes but were not detected in cattle (Fig 4; S1 Appendix).

In the present study, 25 species of the methanogens were reported in the rumen of cattle; while there were 20 species detected in the buffaloes. Among the species, *Methanobrevibacter gottschalkii* had the highest abundance (55-57%) in both the host species. There was no significant (P= 0.869) difference in the distribution between the cattle and buffaloes. The relative abundance of *Methanobrevibacter wolinii* despite the limited representation in the rumen varied significantly (P=0.049) between the two host species. Their distribution was significantly greater in cattle as compared to the buffaloes. Though the abundance of each of *Methanobacterium* *formicicum, Methanobacterium beijingense, Methanobacterium sp., Methanobacterium movens, group 8, Methanosarcina barkeri* and *Methanosarcina sp.* was very limited, they were all, nevertheless, exclusively detected in the rumen of cattle. Similarly, *Group9 sp. CH1270* and *Methanocella arvoryzae* methanogens were exclusively identified in the buffaloes (Fig 4; S1 Appendix).

**Spatial components of biodiversity in the hosts**

The comparative archaeal distribution in cattle and buffaloes studied using Shannon index (alpha diversity) and Bray-Curtis (beta diversity) is presented in Fig 5. There was no significant difference (P=0.66) in the alpha diversity between host species. However, the overall rumen methanogens diversity between cattle and buffaloes was significant (P= 0.015). Betadisper test was not significant indicating a multivariate homogeneity of host dispersion (P=0.648). Bray-Curtis beta diversity analysis indicated a significant difference in rumen methanogens composition between the host species (P=0.01).

**Fig 5** (a) Alpha diversity and (b) Beta diversity of the rumen methanogens in cattle and buffaloes

**Core methanogens analysis**

The methanogens constituted to the core archaeome at the genus level and it was similar in cattle and buffaloes. However, *Methanomicrobium* was exclusively identified as a part of the archaeal core microbiome in cattle (Fig 6a and 6b). At the species level, *Methanobrevibacter gottschalkii clade, Methanobrevibacter ruminantium clade, Methanobacterium alkaliphilum,* and *Methanosphaera sp. ISO3-F5* composed the core methanogens microbiome in both host species. The methanogens such as *Methanomicrobium mobile* and *Methanobrevibacter wolinii* were exclusively present in the archaeal core microbiome in cattle (Fig 6c). On the other hand, *Methanobrevibacter smithii* exclusively represented the methanogens core microbiome in buffaloes (Fig 6d).

**Fig 6** Rumen archaea representing the core microbiome at 50% minimum prevalence in (a) Cattle at genus level (b) buffalo at genus level (c) cattle at species level (d) buffaloes at species level. The colour gradient indicates variability in prevalence

**Discussion**

There is dearth of literature comparing the methane emissions between cattle and buffaloes fed on the same diet and maintained under similar environmental conditions. Our results revealed that the daily methane emissions were significantly greater in cattle than in buffaloes. However, this difference in daily methane emissions was attributed to the significantly greater dry matter intake and body weight in cattle (BW 538 kg; 10.5 kg DMI) as compared to buffaloes (BW 284 kg; 6.86 kg DMI). These results are in good agreement with a previous study [43], where a significant difference in enteric methane emission due to higher dry matter intake and body weight between cattle and buffaloes was reported. However, our findings show that methane yields in cattle and buffaloes were not different.

This study did not detect any statistical difference in the concentrations of ammonia or total volatile fatty acids in the ruminal fluid of cattle and buffaloes, although there were some substantial numerical differences. Similarly, except for iso-butyrate, there were no differences in the molar proportions of individual volatile fatty acids in the ruminal fluid of cattle and buffaloes. The greater dry matter intake of cattle compared to that of buffaloes can be attributed to the greater body weight of cattle compared to buffaloes. Both total protozoa and *Entodiniomorphs* numbers were significantly smaller in cattle as compared to buffaloes (Table 1). This is in agreement with the findings of Jabari *et al.* (2014). The greater dry matter intake in buffaloes could be attributed to the more protozoal concentration that leads to a faster rate of fermentation as compared to cattle [44].

Archaea belong to the domain *Euryarchaeota* represents 3-5% of the rumen microbiota [7,8]; nevertheless, they have a major role in maintaining a low H_2_ pressure within the desirable limits [9,10]. The ruminal methanogen community have a complex syntrophic association with the ruminal microbiota and have diverse substrate requirements for producing methane. Despite being an important integral component of microbiota, only <10% of the methanogens so far have been successfully isolated from the rumen [45]. Understanding the rumen archaeal community in different host animals is a pre-requisite for developing effective methane mitigation strategies. With the development of cultivation-independent techniques, bioinformatics tools, and precise taxonomic framework [37,46], the species-level identification of the archaea to a quite large extent has now become possible.

It has been reported that the rumen microbiota could be affected by the host [8,47,48], diet, and geographical locations [8]. A remarkable difference in the rumen microbiota composition was observed between hosts [49] and breeds fed on a similar diet [50]. In a global study, Henderson *et al.* (2015) demonstrated that diet and host as compared to the geographical locations have a major impact on the rumen microbiome. Their study in cattle from Ontario and Prince Edward islands demonstrated the influence of geographical locations and diet on the diversity of methanogens [50]. Their findings confirmed that the rumen archaeal community are less diversified than the bacteria and a major fraction of the archaea remain invariate across geographical locations [51]. More recently, [52] established that the geographical region and host influence the archaeal community composition. The multiple factors (diet, host, geographical locations) were confounded in most of these studies and due to the variability of more than one factor, it is very difficult to clearly separate the impact of host or diet or geographical locations on the rumen methanogens community structure. To overcome the confounding effect, the present study reports the effect of host species on the rumen methanogens diversity while two other important factors diet and geographical locations were kept constant. While the present study was conducted with a single diet, and we speculate results might differ with other diets, our study indicates the contribution of the host species on rumen methanogens community structure and therefore, devising the methane mitigation strategies accordingly.

In the present study, the rumen archaeal community in both cattle and buffaloes was dominated by the methanogens affiliated to the phylum *Euryarchaeota*. However, methanogens associated with the phylum *Crenarchaeota* were also identified that constituted <1% of total archaea in the rumen. These results are in good agreement with the previous reports [53–55], where *Crenarchaeota* methanogens were previously identified in the rumen. *Sulfolobales* are morphologically and physiologically distinguished archaea, belong to the phylum *Crenarchaeota,* and occurs mainly in extreme thermoacidophilic ecosystems [56]. *Sulfolobales* have been previously identified in the solfataric fields all around the world. In the present study *Sulfolobus thuringiensis* was identified in both the host species. The abundance frequency of *Sulfolobus thuringiensis* is corroborated by [57], who also reported that *Sulfolobus thuringiensis* are distributed up to 0.1% of total archaea in dairy calves. To the best of our knowledge, this is only the second report in the world and first from India confirming the presence of *Sulfolobus thuringiensis* in livestock. However, the methanogenic capabilities of *Sulfolobus thuringiensis,* contribution to the rumen methanogenesis and syntrophic association with other ruminal microbes are not known and need to be explored.

Despite the greatest (72-73%) distribution of *Methanobacteriales* in the rumen, their distribution was similar in both the cattle and buffaloes. Among the genera, *Methanobrevibacter* irrespective of the host species represented the largest fraction (66-68%) of the archaea and they were similarly distributed between cattle and buffaloes. These results are in congruence with previous studies that reported the dominance of *Methanobrevibacter* in both cattle [58,59] and buffaloes [60,61]. A meta-analysis, [62] concluded that the methanogens associated with the genus *Methanobrevibacter* are similarly distributed in cattle and buffaloes. Further, similar distributions of *Methanobrevibacter* in cattle and buffaloes were reported in a recent country-wide study by [52]. The above studies reporting the similar distribution of *Methanobrevibacter* were either conducted separately in cattle and buffaloes or both the species were fed on different diets or having uncontrolled environmental conditions prevailing in different geographical regions; hence, these previous studies did not reveal the actual impact of the hosts on the distribution of rumen methanogens. Our results are consistent with [49], who also reported the dominance of *Methanobrevibacter* in both the Jersey cattle and water buffaloes fed on a similar diet comprising corn silage and concentrate-based diet in the same environmental conditions.

At the species level, *Methanobrevibacter gottschalkii* was the most abundant methanogen and the host species did not have any significant impact on their distribution. The results on the dominance of *Methanobrevibacter gottschalkii* in the rumen archaeal community are in good agreement with previous reports [8,63]. On the contrary, a significant difference in the abundance of *Methanobrevibacter gottschalkii* between cattle and buffaloes has been previously reported by [52]. This disagreement for the distribution of *Methanobrevibacter gottschalkii* between two hosts can be attributed to the confounding effect of multiple factors that remain uncontrolled in their study. Despite the limited distribution, the abundance of *Methanobrevibacter wolinii*, a hydrogenotrophic methanogen was significantly greater (P=0.049) in cattle. Earlier studies also confirmed the presence of *Methanobrevibacter wolinii* in the rumen [64–66]. However, the contribution of *Methanobrevibacter wolinii* to ruminal methanogenesis has not yet been explored and needs further investigation to confirm the impact of distribution pattern on the methane production between cattle and buffaloes.

*Methanomicrobiales* distribution in our study was <4% of the total archaea in both cattle and buffaloes and the abundance frequency was consistent with the global data sets [8,53,65]. However, few previous studies have reported an unprecedented greater abundance of *Methanomicrobium* in cattle [21,22] or buffaloes [67–69]. This disagreement for the distribution of *Methanomicrobiales* methanogens could be attributed to the different DNA isolation methods, primer sets, and animal diets. The comparable distribution of the *Methanomicrobium* between cattle and buffaloes revealed that their distribution was somewhat consistent and was not affected by the host animal under similar diet and environmental conditions.

Three species of the methanogens associated the genus *Methanocella* have been previously reported [70] and isolated from soil of rice fields [71]; however, only one species *Methanocella arvoryzae,* was identified in our study in the buffaloes rumen and that was at a very low frequency (0.002%). *Methanocellales* are non-motile, irregular rod shaped and obligate hydrogenotrophes that primarily utilize H_2_ as an electron donor; however, some species use formate as an electron donor. The low distribution of *Methanocellales* in the buffaloes indicated that they are unlikely to significantly contribute to rumen methanogenesis.

*Methanomassiliicoccales* are methylotrophic methanogens that utilize methanol and methylamines for producing methane [72–74]. *Methanomassiliicoccales* were previously identified in the rumen [18,75,76]. Earlier, the *Methanomassiliicoccales* were placed under the RCC (rumen cluster C) and *Methanoplasmatales* [78–80]. The distribution frequency of *Methanomassiliicoccales* in this study is consistent with [46]. At the genus level, the abundances of group 9, group 10 and group 4 were similar and not affected by the host species. Jin *et al.* [72] from a study in goats, concluded that the high grain diet feeding stimulates the higher abundances of group 10 and group 4 methanogens; while high hay diet led to a higher abundance of group 9 methanogens at the genus level. A decrease in rumen pH due to high grain feeding is possibly a general cause for the stimulation or suppression of a particular type of methylotrophs in the rumen [81]. Therefore, the relative abundance of *Methanomassiliicoccales* appears to be affected by the diet rather than the host. The *Methanomassiliicoccales* may have specific properties that allowed their survival or inhibition during variable pH, and this requires investigation. In our study, group 8 methylotrophs were exclusively identified in the cattle. The greater distribution of group 8 methanogens in cattle compared to their distribution in buffaloes has recently been confirmed [52]. This difference should be taken into account while developing strategies for methane mitigation from the cattle and buffaloes. The methane producing capabilities of *Methanomassiliicoccales* have not yet been explored and this warrants further investigation to establish their contribution to the rumen methanogenesis.

*Methanosarcinales* grow on a broad range of substrates such as H_2_ and, CO_2_, methanol, methylamines and acetate. They are the only methanogens that possess cytochromes [82]. Methanogens associated to the *Methanosarcinales* are mostly coccoid shaped, but without motility [11] and this is the only methanogen order capable of performing acetoclastic methanogenesis [83]. In this study, although *Methanosarcinales* were distributed at very small frequencies (<0.5%) in both the cattle and buffaloes, their abundance was significantly greater (P=1e^-6^) in cattle compared to in buffaloes. *Methanosarcinales* have been previously identified in both cattle [84,85] and buffaloes [67]. In the present study, the low distribution of *Methanosarcinales* in both host species may be attributed to their developmental age (adult), as this group of methanogens have been reported to perform methanogenesis exclusively in the young rumen, while hydrogenotrophic methanogens are prominently involved in methanogenesis in the mature rumen [86].

In this study, the rod shape, non-motile *Methanosaeta* *harundinacea*, an acetoclastic methanogen was exclusively detected (0.01%) in the rumen of cattle. This methanogen was isolated from a UASB reactor [87]. Although we have not differentiated the distribution of *Methanosaeta* between solid and liquid phases in this study; nevertheless, their strict acetoclastic nature makes it likely that they are more abundant in the liquid phase [88].

**Conclusions**

It is concluded that the methane yields of cattle and buffaloes fed on the same diet did not differ. Methanogens associated to the phylum *Euryarchaeota* dominated the community in both host species and *Crenarchaeota* (*Sulfolobus thuringiensis)* represented a limited fraction of the archaeal community. *Methanobrevibacter* was the most prominent genus of methanogens; however, they are distributed similarly in both host species. *Methanobrevibacter gottschalkii* despite the highest abundance did not show any host-specific difference. The relative abundances of *Methanobrevibacter wolinii* and *Methanosarcinales* were significantly greater in cattle than in buffaloes. There were a few methanogens identified exclusively either in cattle (*Methanosaeta* and group 8) or in buffaloes (*Methanocella*). Thus, it is evident from the study that when the diet and environmental conditions are same, the host has a limited influence on the rumen archaeal community structure. Accordingly, we speculate that methane mitigation strategies developed in either of the hosts should be effective in the other one. However, further studies may be useful to confirm these findings with other diets and in other geographical locations.

**Acknowledgements**

The authors are thankful to the International Livestock Research Institute (ILRI), Nairobi and Indian Council of Agricultural Research, New Delhi for funding a collaborative research project on *Methane Emission and its Mitigation* through window-III of CGIAR. we thankfully acknowledge the support received from the Project Coordinator, ILRI, South Asia Office, New Delhi and the Director, ICAR-NIANP, Bangalore for providing scientific inputs and logistics supports in carrying out the research.

**References**

1.	Yusuf RO, Noor ZZ, Abba AH, Hassan MAA, Din MFM. Methane emission by sectors: A comprehensive review of emission sources and mitigation methods. Renew Sustain Energy Rev. 2012;16: 5059–5070. doi:10.1016/j.rser.2012.04.008

2.	Saunois M, Jackson RB, Bousquet P, Poulter B, Canadell JG. The growing role of methane in anthropogenic climate change. Environ Res Lett. 2016;11. doi:10.1088/1748-9326/11/12/120207

3.	Government of India. 20th Livestock census: provisional key results. Department of Animal Husbandry and Dairying, Ministry of Fisheries, Animal Husbandry {\&} Dairying, Govt. of India. 2019. Available: http://www.dahd.nic.in/division/provisional-key-results-20th-livestock-census

4.	Bhatta R, Malik PK, Kolte AP, Suresh KP. Assessment of enteric methane emission from Indian livestock: a new approach. In: Sejian V, Isloor S, Rahman SA, Bhatta R, editors. 7th Pan Commonwealth Veterinary Conference. Bengaluru: Commonwealth Veterinary Association(Asia); 2019. pp. 101–103.

5.	Guan H, Wittenberg KM, Ominski KH, Krause DO. Efficacy of ionophores in cattle diets for mitigation of enteric methane. J Anim Sci. 2006;84: 1896–1906. doi:10.2527/jas.2005-652

6.	Johnson KA, Johnson DE. Methane emissions from cattle. J Anim Sci. 1995;73: 2483–2492. doi:10.2527/1995.7382483x

7.	Yanagita K, Kamagata Y, Kawaharasaki M, Suzuki T, Nakamura Y, Minato H. Phylogenetic analysis of methanogens in sheep rumen ecosystem and detection of methanomicrobium mobile by fluorescence in situ hybridization. Bioscience, Biotechnology and Biochemistry. 2000. pp. 1737–1742. doi:10.1271/bbb.64.1737

8.	Henderson G, Cox F, Ganesh S, Jonker A, Young W, Janssen PH, et al. Rumen microbial community composition varies with diet and host, but a core microbiome is found across a wide geographical range. Sci Rep. 2015;5. doi:10.1038/srep14567

9.	Malik PK, Bhatta R, Soren NM, Sejian V, Mech A, Prasad KS, et al. Feed-based approaches in enteric methane amelioration. In: Malik PK, Bhatta R, Takahashi J, Kohn RA, Prasad CS, editors. Livestock production and climate change. CABI; 2015. pp. 336–359. doi:10.1079/9781780644325.0336

10.	Gagen EJ, Denman SE, McSweeney CS. Acetogenesis as an alternative to methanogenesis in rumen. In: Malik PK, Bhatta R, Takahashi J, Kohn RA, Prasad CS, editors. Livestock production and climate change. CABI; 2015. pp. 292–303. doi:10.1079/9781780644325.0183

11.	Balch WE, Fox GE, Magrum LJ, Woese CR, Wolfe RS. Methanogens: reevaluation of a unique biological group. Microbiol Rev. 1979;43: 260–296. doi:10.1128/mmbr.43.2.260-296.1979

12.	Hook SE, Wright ADG, McBride BW. Methanogens: Methane producers of the rumen and mitigation strategies. Archaea. 2010;2010: 50–60. doi:10.1155/2010/945785

13.	Greening C, Ahmed FH, Mohamed AE, Lee BM, Pandey G, Warden AC, et al. Physiology, Biochemistry, and Applications of F420- and Fo-Dependent Redox Reactions. 2016;80: 451–493. doi:10.1128/MMBR.00070-15.Address

14.	Hungate RE. SYMPOSIUM. Microbes of nutritional importance in the alimentary tract. Proceedings of the Niutrition Society; 1984. pp. 1–11.

15.	Zhou M, Hernandez-Sanabria E, Luo Guan L. Characterization of variation in rumen methanogenic communities under different dietary and host feed efficiency conditions, as determined by PCR-denaturing gradient gel electrophoresis analysis. Appl Environ Microbiol. 2010;76: 3776–3786. doi:10.1128/AEM.00010-10

16.	St-Pierre B, Wright ADG. Diversity of gut methanogens in herbivorous animals. Animal. 2013;7: 49–56. doi:10.1017/S1751731112000912

17.	Huang XD, Tan HY, Long R, Liang JB, Wright ADG. Comparison of methanogen diversity of yak (Bos grunniens) and cattle (Bos taurus) from the Qinghai-Tibetan plateau, China. BMC Microbiol. 2012;12. doi:10.1186/1471-2180-12-237

18.	Huang XD, Martinez-Fernandez G, Padmanabha J, Long R, Denman SE, McSweeney CS. Methanogen Diversity in Indigenous and Introduced Ruminant Species on the Tibetan Plateau. Archaea. 2016;2016. doi:10.1155/2016/5916067

19.	von Keyserlingk GE, Mathison GW. The effect of ruminal escape protein and ambient temperature on the efficiency of utilization of metabolizable energy by lambs. J Anim Sci. 1993;71: 2206–2217. doi:10.2527/1993.7182206x

20.	Henderson G, Cox F, Ganesh S, Jonker A, Young W, Janssen PH. Rumen microbial community composition varies with diet and host, but a core microbiome is found across a wide geographical range. Sci Rep. 2015;5: 14567. doi:10.1038/srep14567

21.	Shin EC, Choi BR, Lim WJ, Hong SY, An CL, Cho KM, et al. Phylogenetic analysis of archaea in three fractions of cow rumen based on the 16S rDNA sequence. Anaerobe. 2004;10: 313–319. doi:10.1016/j.anaerobe.2004.08.002

22.	Tajima K, Aminov RI, Nagamine T, Matsui M, Nakamura M, Benno Y. Diet dependent shifts in the bacterial population of the rumen revelaed by real time PCR. Appl Environ Microbiol. 2001;67: 2766–2774. doi:10.1128/AEM.67.6.2766

23.	Whitford MF, Teather RM, Forster RJ. Phylogenetic analysis of methanogens from the bovine rumen. BMC Microbiol. 2001;1: 1–5. doi:10.1186/1471-2180-1-5

24.	Wright ADG, Auckland CH, Lynn DH. Molecular diversity of methanogens in feedlot cattle from Ontario and Prince Edward Island, Canada. Appl Environ Microbiol. 2007;73: 4206–4210. doi:10.1128/AEM.00103-07

25.	AOAC. Official Methanods of Analysis of AOAC International. 19th Editi. Gaithersburg, MD: AOAC International; 2012.

26.	Van Soest PJ, Robertson JB, Lewis BA. Methods for dietary fiber, neutral detergent fiber, and nonstarch polysaccharides in relation to animal nutrition. J Dairy Sci. 1991;74: 3583–3597.

27.	Johnson KA, Huyler MT, Westburg HH, Lamb BK, Zimmerman P. Johnson, K. A.; Huyler, M. T.; Westburg, H. H.; Lamb, B. K.; Zimmerman, P. Measurement of methane emissions from ruminant livestock using a SF6 tracer technique. Environ Sci Technol. 1994;28: 359–362.

28.	Pinares-Patiño CS, Clark H. Reliability of the sulfur hexafluoride tracer technique for methane emission measurement from individual animals: An overview. Aust J Exp Agric. 2008;48: 223–229. doi:10.1071/EA07297

29.	Filípek J, Dvořák R. Determination of the volatile fatty acid content in the rumen liquid: Comparison of gas chromatography and capillary isotachophoresis. Acta Vet Brno. 2009;78: 627–633. doi:10.2754/avb200978040627

30.	Conway EJ. Micro-diffusion Analysis and Volumetric Error. 4th edn. London Crossby: Lockwood and Sons Ltd.; 1957.

31.	Kamra DN, Sawal RK, Pathak NN, Kewalramani N, Agarwal N. Diurnal variation in ciliate protozoa in the rumen of black buck (Antilope cervicapra) fed green forage. Lett Appl Microbiol. 1991;13: 165–167. doi:10.1111/j.1472-765X.1991.tb00598.x

32.	Hungate RE. The rumen and its microbes [Internet]. Academic press inc., New York, USA. (1966). New York, USA: Academic press inc.; 1966.

33.	Yu Z, Morrison M. Improved extraction of PCR-quality community DNA from digesta and fecal samples. Biotechniques. 2004;36: 808–812. doi:10.2144/04365st04

34.	Wemheuer B, Wemheuer F, Daniel R. RNA-based assessment of diversity and composition of active archaeal communities in the german bight. Archaea. 2012;2012. doi:10.1155/2012/695826

35.	Takai K, Horikoshi K. Rapid detection and quantification of members of the archaeal community by quantitative PCR using fluorogenic probes. Appl Environ Microbiol. 2000;66: 5066–5072. doi:10.1128/AEM.66.11.5066-5072.2000

36.	Callahan BJ, Sankaran K, Fukuyama JA, McMurdie PJ, Holmes SP. Bioconductor workflow for microbiome data analysis: From raw reads to community analyses [version 1; referees: 3 approved]. F1000Research. 2016;5: 1–48. doi:10.12688/F1000RESEARCH.8986.1

37.	Seedorf H, Kittelmann S, Henderson G, Janssen PH. RIM-DB: A taxonomic framework for community structure analysis of methanogenic archaea fromthe rumen and other intestinal environments. PeerJ. 2014;2014. doi:10.7717/peerj.494

38.	McMurdie PJ, Holmes S. Phyloseq: An R Package for Reproducible Interactive Analysis and Graphics of Microbiome Census Data. PLoS One. 2013;8. doi:10.1371/journal.pone.0061217

39.	Oksanen J, Blanchet FG, Kindt R, Legendre P, Minchin PR, O’hara RB, et al. “Package ‘vegan’.” Community ecology package, version 2, no. 9. 2013. pp. 1–295.

40.	Wickham H. ggplot2. Wiley Interdiscip Rev Comput Stat. 2011;3: 180–185. doi:10.1002/wics.147

41.	Love MI, Huber W, Anders S. Moderated estimation of fold change and dispersion for RNA-seq data with DESeq2. Genome Biol. 2014;15: 1–21. doi:10.1186/s13059-014-0550-8

42.	Lahti L, Shetty S. Microbiome@GitHub. 2012 [cited 31 Jul 2020]. Available: url: http://microbiome.github.com/microbiome

43.	Malik PK. Effect of dietary leguminous fodder on methane and nitrous oxide emission from ruminants. National Dairy Research Institute, Karnal, Hariyana, India. 2006.

44.	Hungate RE, Phillips GD, Hungate DP, MacGregor A. A comparison of the rumen fermentation in european and zebu cattle. J Agric Sci. 1960;54: 196–201.

45.	Kim CC. Identification of rumen methanogens , characterization of substrate requirements and measurement of hydrogen thresholds. 2012; 1–141.

46.	Seedorf H, Kittelmann S, Janssen PH. Few highly abundant operational taxonomic units dominate within rumen methanogenic archaeal species in New Zealand sheep and cattle. Appl Environ Microbiol. 2015;81: 986–995. doi:10.1128/AEM.03018-14

47.	Guan LL, Nkrumah JD, Basarab JA, Moore SS. Linkage of microbial ecology to phenotype: Correlation of rumen microbial ecology to cattle’s feed efficiency. FEMS Microbiol Lett. 2008;288: 85–91. doi:10.1111/j.1574-6968.2008.01343.x

48.	Roehe R, Dewhurst RJ, Duthie CA, Rooke JA, McKain N, Ross DW, et al. Bovine Host Genetic Variation Influences Rumen Microbial Methane Production with Best Selection Criterion for Low Methane Emitting and Efficiently Feed Converting Hosts Based on Metagenomic Gene Abundance. PLoS Genet. 2016;12: 1–20. doi:10.1371/journal.pgen.1005846

49.	Iqbal MW, Zhang Q, Yang Y, Li L, Zou C, Huang C, et al. Comparative study of rumen fermentation and microbial community differences between water buffalo and Jersey cows under similar feeding conditions. J Appl Anim Res. 2018;46: 740–748. doi:10.1080/09712119.2017.1394859

50.	Paz HA, Anderson CL, Muller MJ, Kononoff PJ, Fernando SC. Rumen bacterial community composition in holstein and jersey cows is different under same dietary condition and is not affected by sampling method. Front Microbiol. 2016;7: 1–9. doi:10.3389/fmicb.2016.01206

51.	Hook SE, Wright ADG, McBride BW. Methanogens: Methane producers of the rumen and mitigation strategies. Archaea. 2010;2010. doi:10.1155/2010/945785

52.	Trivedi S, Kumar Malik P, Purushottam Kolte A, Thirumalaisamy G, Shyamili Kumar V, Veerasamy S, et al. Inuence of Host and Geographical Regions on the Rumen Methanogens Diversity in Indian Cattle and Buffaloes. Res Sq. 2020; 1–24. doi:doi.org/10.21203/rs.3.rs-53542/v1

53.	Janssen PH, Kirs M. Structure of the archaeal community of the rumen. Appl Environ Microbiol. 2008;74: 3619–3625. doi:10.1128/AEM.02812-07

54.	Abecia L, Waddams KE, Martínez-Fernandez G, Martín-García AI, Ramos-Morales E, Newbold CJ, et al. An antimethanogenic nutritional intervention in early life of ruminants modifies ruminal colonization by archaea. Archaea. 2014;2014. doi:10.1155/2014/841463

55.	Xue F, Nan X, Li Y, Pan X, Guo Y, Jiang L, et al. Metagenomic insights into effects of thiamine supplementation on carbohydrate-active enzymes’ profile in dairy cows fed high-concentrate diets. Animals. 2019;10: 1–11. doi:10.3390/ani10020304

56.	Quehenberger J, Shen L, Albers SV, Siebers B, Spadiut O. Sulfolobus - A potential key organism in future biotechnology. Front Microbiol. 2017;8: 1–13. doi:10.3389/fmicb.2017.02474

57.	Dias J, Marcondes MI, Noronha MF, Resende RT, Machado FS, Mantovani HC, et al. Effect of pre-weaning diet on the ruminal archaeal, bacterial, and fungal communities of dairy calves. Front Microbiol. 2017;8. doi:10.3389/fmicb.2017.01553

58.	Sirohi SK, Chaudhary PP, Singh N, Singh D, Puniya AK. The 16S rRNA and mcrA gene based comparative diversity of methanogens in cattle fed on high fibre based diet. Gene. 2013;523: 161–166. doi:10.1016/j.gene.2013.04.002

59.	Parmar NR, Pandit PD, Purohit HJ, Nirmal Kumar JI, Joshi CG. Influence of Diet Composition on Cattle Rumen Methanogenesis: A Comparative Metagenomic Analysis in Indian and Exotic Cattle. Indian J Microbiol. 2017;57: 226–234. doi:10.1007/s12088-016-0635-z

60.	Franzolin R, St-Pierre B, Northwood K, Wright A-DG. Analysis of Rumen Methanogen Diversity in Water Buffaloes (Bubalus bubalis) Under. Ecology. 2012;64: 131–139. doi:10.1007/s00248-0

61.	Kumar S, Dagar SS, Agrawal RK, Puniya AK. Comparative diversity analysis of ruminal methanogens in Murrah buffaloes (Bubalus bubalis) in four states of North India. Anaerobe. 2018;52: 59–63. doi:10.1016/j.anaerobe.2018.05.012

62.	Paul SS, Dey A, Baro D, Punia BS. Comparative community structure of archaea in rumen of buffaloes and cattle. J Sci Food Agric. 2017;97: 3284–3293. doi:10.1002/jsfa.8177

63.	Wright ADG, Ma X, Obispo NE. Methanobrevibacter phylotypes are the dominant methanogens in sheep from Venezuela. Microb Ecol. 2008;56: 390–394. doi:10.1007/s00248-007-9351-x

64.	Skillman LC, Evans PN, Naylor GE, Morvan B, Jarvis GN, Joblin KN. 16S ribosomal DNA-directed PCR primers for ruminal methanogens and identification of methanogens colonising young lambs. Anaerobe. 2004;10: 277–285. doi:10.1016/j.anaerobe.2004.05.003

65.	Zhou M, Hernandez-Sanabria E, Le LG. Assessment of the microbial ecology of ruminal methanogens in cattle with different feed efficiencies. Appl Environ Microbiol. 2009;75: 6524–6533. doi:10.1128/AEM.02815-08

66.	Leahy SC, Kelly WJ, Li D, Li Y, Altermann E, Lambie SC, et al. The Complete genome sequence of Methanobrevibacter sp. ABM4. Stand Genomic Sci. 2013;8: 215–227. doi:10.4056/sigs.3977691

67.	Singh KM, Pandya PR, Parnerkar S, Tripathi AK, Rank DN, Kothari RK, et al. Molecular identification of methanogenic archaea from surti buffaloes (Bubalus bubalis), reveals more hydrogenotrophic methanogens phylotypes. Brazilian J Microbiol. 2011;42: 132–139. doi:10.1590/S1517-83822011000100017

68.	Singh KM, Tripathi AK, Pandya PR, Parnerkar S, Rank DN, Kothari RK, et al. Methanogen diversity in the rumen of Indian Surti buffalo (Bubalus bubalis), assessed by 16S rDNA analysis. Res Vet Sci. 2012;92: 451–455. doi:10.1016/j.rvsc.2011.03.022

69.	Chaudhary PP, Sirohi SK. Dominance of Methanomicrobium phylotype in methanogen population present in Murrah buffaloes (Bubalus bubalis). Lett Appl Microbiol. 2009;49: 274–277. doi:10.1111/j.1472-765X.2009.02654.x

70.	Sakai S, Imachi H, Hanada S, Ohashi A, Harada H, Kamagata Y. Methanocella paludicola gen. nov., sp. nov., a methane-producing archaeon, the first isolate of the lineage “Rice Cluster I”, and proposal of the new archaeal order Methanocellales ord. nov. Int J Syst Evol Microbiol. 2008;58: 929–936. doi:10.1099/ijs.0.65571-0

71.	Lü Z, Lu Y. Methanocella conradii sp. nov., a thermophilic, obligate hydrogenotrophic methanogen, isolated from chinese rice field soil. PLoS One. 2012;7. doi:10.1371/journal.pone.0035279

72.	Jin W, Cheng Y, Zhu W. The community structure of Methanomassiliicoccales in the rumen of Chinese goats and its response to a high-grain diet. J Anim Sci Biotechnol. 2017;8: 1–10. doi:10.1186/s40104-017-0178-0

73.	Lang K, Schuldes J, Klingl A, Poehlein A, Daniel R, Brune A. New mode of energy metabolism in the seventh order of methanogens as revealed by comparative genome analysis of “Candidatus Methanoplasma termitum.” Appl Environ Microbiol. 2015;81: 1338–1352. doi:10.1128/AEM.03389-14

74.	Söllinger A, Schwab C, Weinmaier T, Loy A, Tveit AT, Schleper C, et al. Phylogenetic and genomic analysis of Methanomassiliicoccales in wetlands and animal intestinal tracts reveals clade-specific habitat. FEMS Microbiol Ecol. 2016;92: 1–12. doi:10.1093/femsec/fiv149

75.	Pol A, Demeyer DI. Fermentation of methanol in the sheep rumen. Appl Environ Microbiol. 1988;54: 832–834. doi:10.1128/aem.54.3.832-834.1988

76.	Martz FA, Payne CP, Matches AG, Belyea RL, Warren WP. Forage Intake, Ruminal Dry Matter Disappearance, and Ruminal Blood Volatile Fatty Acids for Steers in 18 and 32°C Temperatures. J Dairy Sci. 1990;73: 1280–1287. doi:10.3168/jds.S0022-0302(90)78793-0

77.	Poulsen M, Schwab C, Borg Jensen B, Engberg RM, Spang A, Canibe N, et al. Methylotrophic methanogenic Thermoplasmata implicated in reduced methane emissions from bovine rumen. Nat Commun. 2013;4. doi:10.1038/ncomms2432

78.	Dridi B, Raoult D, Drancourt M. Matrix-assisted laser desorption/ionization time-of-flight mass spectrometry identification of Archaea: Towards the universal identification of living organisms. Apmis. 2012;120: 85–91. doi:10.1111/j.1600-0463.2011.02833.x

79.	Iino T, Tamaki H, Tamazawa S, Ueno Y, Ohkuma M, Suzuki KI, et al. Candidatus methanogranum caenicola: A novel methanogen from the anaerobic digested sludge, and proposal of Methanomassiliicoccaceae fam. nov. and Methanomassiliicoccales ord. nov., for a methanogenic lineage of the class Thermoplasmata. Microbes Environ. 2013;28: 244–250. doi:10.1264/jsme2.ME12189

80.	Paul K, Nonoh JO, Mikulski L, Brune A. “Methanoplasmatales,” thermoplasmatales-related archaea in termite guts and other environments, are the seventh order of methanogens. Appl Environ Microbiol. 2012;78: 8245–8253. doi:10.1128/AEM.02193-12

81.	Lana RP, Russell JB, Van Amburgh ME. The Role of pH in Regulating Ruminai Methane and Ammonia Production. J Anim Sci. 1998;76: 2190–2196. doi:10.2527/1998.7682190x

82.	Thauer RK, Kaster AK, Seedorf H, Buckel W, Hedderich R. Methanogenic archaea: Ecologically relevant differences in energy conservation. Nat Rev Microbiol. 2008;6: 579–591. doi:10.1038/nrmicro1931

83.	Kendall MM, Boone DR. The Order Methanosarcinales. Third Edit. In: Falkow S, Rosenberg E, Schleifer K-H, Stackebrandt E, editors. The Prokaryotes. Third Edit. Minneapolis: Springer USA; 2006. pp. 244–256.

84.	Jarvis GN, Strömpl C, Burgess DM, Skillman LC, Moore ERB, Joblin KN. Isolation and identification of ruminal methanogens from grazing cattle. Curr Microbiol. 2000;40: 327–332. doi:10.1007/s002849910065

85.	Patterson JA, Hespell RB. Trimethylamine and methylamine as growth substrates for rumen bacteria andMethanosarcina barkeri. Curr Microbiol. 1979;3: 79–83.

86.	Friedman N, Jami E, Mizrahi I. Compositional and functional dynamics of the bovine rumen methanogenic community across different developmental stages. Environ Microbiol. 2017;19: 3365–3373. doi:10.1111/1462-2920.13846

87.	Ma K, Liu X, Dong X. Methanosaeta harundinacea sp. nov., a novel acetate-scavenging methanogen isolated from a UASB reactor. Int J Syst Evol Microbiol. 2006;56: 127–131. doi:10.1099/ijs.0.63887-0

88.	Mahmood M, Khiaosa-Ard R, Zebeli Q, Petri RM. Betaine Modulates Rumen Archaeal Community and Functioning during Heat and Osmotic Stress Conditions in Vitro. Archaea. 2020;2020. doi:10.1155/2020/8875773

**Supporting information**

S1 Appendix: Details of reads, relative abundance and statistical analysis

S1 Table: Chemical composition of the diet ingredients
