# Supplementary material for: Comparison of enteric methane yield and diversity of ruminal methanogens in cattle and buffaloes fed on the same diet
Source: PLoS One. 2021 Aug 11;16(8):e0256048. doi: 10.1371/journal.pone.0256048 (PMC8357158; doi:10.1371/journal.pone.0256048)
Supplement: Attachment — Submitted filename: Response to Reviewers.docx [file pone.0256048.s005.docx]

**Response to Editor and Reviewers**

We would like to thank both the reviewers for sparing their valuable time during the COVID pandemic. The comments and suggestions provided by both the reviewers are very constructive and relevant which improved the manuscript quality substantially. This is to certify that all the suggestions have been incorporated in the revised manuscript. We are also thankful to the Editor for providing the inputs to improve the manuscript quality.

The response to the comments of reviewers and editors are appended below.

**Editor’s comment**

| S.N. | Comments/instructions | Response |
| --- | --- | --- |
| 1 | A marked-up copy of your manuscript that highlights changes made to the original version. You should upload this as a separate file labelled 'Revised Manuscript with Track Changes'. | Manuscript highlighting the changes is created separately and given the name ‘Manuscript with Track Changes |
| 2 | An unmarked version of your revised paper without tracked changes. You should upload this as a separate file labelled 'Manuscript'. | A clean manuscript is created without track changes and named as ‘Manuscript’ |
| 3 | If you would like to make changes to your financial disclosure, please include your updated statement in your cover letter. | Updated financial disclosure has been provided in the covering letter |
| 4 | Manuscript should meets PLOS ONE's style requirements, including those for file naming | Manuscript is prepared by strictly following the PLOS ONE style |
| 5 | Amend your current ethics statement to confirm that your named ethics committee specifically approved this study. Once you have amended this/these statement(s) in the Methods section of the manuscript, please add the same text to the “Ethics Statement” field of the submission form (via “Edit Submission”). | Ethics Statement is amended and updated in the materials and methods. It is also provided to the field of submission (Edit Submission) |
| 6 | While revising your submission, please upload your figure files to the Preflight Analysis and Conversion Engine (PACE) digital diagnostic tool. | Figure files were uploaded to the PACE and converted figures are now provided for the manuscript |

**Reviewer # 1**

| S.N. | Comments/instructions | Response |
| --- | --- | --- |
| 1 | The English expression should be substantially improved. In this regard, suggested edits are shown by track changes in the attached file. | Thanks for the suggestion and editing the manuscript. The language has been has been corrected wherever suggested or required. |
| 2 | Some more detail and perhaps recalculation of methane production is needed as it seems an inappropriate calculation was used. Indeed, the reported methane yields for cattle were much smaller than the usual value of about 20 g CH4/kg DMI. Recalculation of methane yields using the equation of Williams et al is likely to produce the expected values. | Thanks for the pointing out less methane yield in both cattle and buffaloes. The lesser methane yield could be a result of the conventional method of calculation (Methane/DMI) used in the manuscript. As per suggestion, we have adopted the Williams et al. 2019 methodology and recalculated the methane yield. The methane yield is 23-24 g/kg DMI. This is in agreement with the previous reports. The methane emission and Methane yield data is now included in Table 1. |
| 3 | Standard errors of means or standard errors of difference should be included in Table 1. | Standard error of means (SEM) has been included in Table 1 |
| 4 | A small number of statements and conclusions are not justified based on the findings of this experiment. Alternative acceptable statements are listed in the attached file. | We are thankful for the valuable suggestions and modifying the manuscript, which substantially improved the quality. The statements have been modified as suggested. |

**Reviewer # 2**

| S.N. | Comments/instructions | Response |
| --- | --- | --- |
| 1 | ‘rumen fluid’ should be ‘ruminal fluid’ | Wherever applicable, rumen fluid is replaced with ruminal fluid |
| 2 | Suggestion for using methane metrics as appear in previously published reports i.e.  Methane emission – the mass of methane emitted per day, g/d Methane yield – the mass of methane emitted per unit of dry matter intake, g/kg DMI | The methane metrics has been presented as suggested. Methane emission- (g/d)  Methane yield (g/kg DMI) |
| 3 | **Title - You report that methane emission was different between cattle and buffalo due to different feed intakes. You use methane yield in the results, so that should be used here. | Enteric Methane Yield has been used in the title |
| 4 | **Introduction - The introduction is littered with poor English expressions. I have noted some, but others still exist. | Thanks a lot for the editing. Modifications have been made wherever necessary and irrelevant content has been deleted from the introduction part |
| 5 | The importance of cattle and buffalo is not explained. I can see that half the world’s buffalo are in India, but so what? You need to explain why you are focusing on cattle and buffalo. | Importance of cattle and buffalo in terms of milk production along with reference is now included in the introduction |
| 6 | Methane is a key part of the title and abstract but is not listed in the objectives. | Comparison of Methane Yield between two host species is incorporated as one of the objectives |
| 7 | I would like to see a hypothesis. Perhaps ‘We hypothesised that there would be no difference in the methane yield or microbial population demographics between cattle and buffalo.’ | Complied |
| 8 | L69 - The reference used is from 2016, making it 5 years out of date. Present data should be available from one of the atmospheric monitoring organisations. For example, National Oceanic and Atmospheric Administration, or CSIRO. | As per suggestion, methane concentration and reference have been changed. A latest reference of NOAA 2021 has been used in the introduction |
| 9 | L69 - Tg is a SI unit so does not need to be explained | Agreed and deleted word teragram |
| 10 | L69 to 71 - These statements should be referenced | Reference is included |
| 11 | L72 - which country? 13% of what population of cattle? | Country name is introduced, the % is now clarified. It is the % of global cattle population |
| 12 | L75 - suggest ‘Methanogenesis is a major sink for hydrogen in the rumen. However, methane has an embodied energy of XXX kJ/g resulting in the methane emitted by cattle accounting for 2 – 12% of gross energy intake.’ | Thanks for editing the statement. It is very well taken |
| 13 | L79 to 85 - This reads as a list of things. There is no logical progression to the argument. | The paragraph has been deleted |
| 14 | L87 - environmental conditions cannot report anything. I suspect you mean ‘Environmental conditions have also been reported to influence the ruminal archaeal community,’ | Agreed. The statement is modified |
| 15 | L88 - Suggest ‘In a global study [20], Methanobrevibacter gottschalkii …’ | Complied |
| 16 | L107 - which institute? | Institute name is incorporated |
| 17 | L116 - How were the animals arranged in the shed? Williams et al 2011 (doi: 10.1071/AN15365) have shown that background concentration of gases varies within a building, even when completely open on one side. | Type of shed, animal arrangements in the shed and background sampling details have been added to the materials and methods |
| 18 | L121 - and elsewhere – I suggest using g/kg instead of parts. EG: maize grain (320 g/kg) | Word ‘parts’ is replaced with g/kg for each ingredient of the concentrate mixture |
| 19 | L123 - ‘basal’ is unnecessary, there is only one diet. | Deleted |
| 20 | L127 - ‘S1 Table’ should be ‘Table S1’ | Table numbering is as per Journal |
| 21 | L130 to 139 This description is inadequate. I cannot replicate your work from the description given. References 27 and 28 do not tell me how the technique was implemented. [27] used stainless steel spheres to sample gas for up to 6 hours, [28] does not describe an implementation of the SF6 technique. I suggest you see chapter Berndt, A, Boland, TM, Deighton, MH, Gere, JI, Grainger, C, Hegarty, RS, Iwaasa, AD, Koolaard, JP, Lassey, KR, Luo, D, Martin, RJ, Martin, C, Moate, PJ, Molano, G, Pinares-Patiño, C, Ribaux, BE, Swainson, NM, Waghorn, GC, Williams, SRO (2020) 'Guidelines for use of sulphur hexafloride (SF6) tracer technique to measure enteric methane emissions from ruminants.' (New Zealand Agricultural Greenhouse Gas Research Centre: New Zealand). | Agreed, it is now elaborated and hanging of canister, background sampling, SF6 release rate and dilution have been included in the methodology.  We really appreciate your efforts in pointing out the issue pertaining to the SF6 method. Since 2015, we are following the Berndt et al. (2014) methodology for the SF6; however, so far we were using Johnson reference as he developed the technique. The reference and formula used for methane calculation was exactly as per Berndt et al. Changes in the reference and formula have been made. |
| 22 | Given the animals were enclosed in a shed, details of how background gas samples were collected is critical. See Williams et al 2011 (doi: 10.1071/AN15365) | The shed and background gas samples collection details are provided in the materials and methods section |
| 23 | There is no indication of the extent of dilution. Were the collection vessels pressurized with nitrogen, and to what pressure. This dilution also needs to be corrected for. Not all collection vessels will be at the same residual vacuum, so the dilution will be different for each sample. For an example of how to do the correction when the canisters are still under vacuum after dilution see Moate et al 2020 (doi: 10.3390/ani10060976). Also, the equation presented is insufficient. See equation 2 in Williams et al 2011 (doi: 10.1016/j.anifeedsci.2011.08.013) for a complete expression of the calculation. | The dilution details for the gas samples with pure N2 are provided in materials and methods. Williams et al. reference is also incorporated. |
| 24 | L147 – 148. The description of the collection of ruminal fluid is insufficient to allow me to repeat the process. | The process has been elaborated |
| 25 | L150 - repeats the previous sentence. | The repetition has been removed |
| 26 | L151 to 153 - How were the samples prepared? Acidified, stained, frozen? | Sample processing for rumen fermentation parameters is elaborated |
| 27 | L158 the description of the processes are inadequate. What detector temperatures were used in your work. what carrier gas, at what flow and pressure? And what was the specification of the column? I would expect to see the model and manufacturer details. | Gas chromatograph conditions are included in the methodology |
| 28 | Was the method for NH3-N exactly as per [30] or were there some implementation details that were different during this analysis? | Yes, we have used the exact protocol described by Convay (1957) |
| 29 | L199 - ‘vegan’ this should be identified as a version of R software. | Incorporated |
| 30 | L204 - suggest ‘adonis function’ be identified as part of R | Incorporated |
| 31 | There is no mention of how the methane data were analysed. | It is mentioned in the methane emission section of materials and methods |
| 32 | higher is used when greater is meant. | Higher is replaced with greater at all the instances |
| 33 | p values – please state the actual p values in the text instead of simply thresholds. | complied |
| 34 | L222 - Once methane yield is defined in the introduction it can be used here. ‘However, there was no difference in methane yield between cattle (13.4 g/kg DMI) and buffaloes (13.5 g/kg DMI).’ Also, these values are much lower than those commonly reported by others. | Methane yield is recalculated using the methodology of Williams et al. (2019). The revised figures are in agreement with the previous reports |
| 35 | L231 - TVFA – true, but I note that the TVFA concentration in cattle is 1.6 time greater than in buffalo | The greater concentration of TVFA in cattle was due to the more dry matter intake in cattle than the buffaloes (10.5 Vs 6.86) |
|  | L234 greater in buffalo compared to what? | Corrected |
| 36 | L235 While I note that there is no statistical difference, the concentration of Holotrichs in cattle were only 75% of that in buffalo. | It could be due to sample to sample variation within the group |
| 37 | Table 1 There is no indication of error in the results but there should be | Standard error of mean has been incorporated in Table 1 |
| 38 | L242 ‘reads were’ | Modified |
| 39 | Figure 2 is useless to me. So it shows OTU, but of what? | Figure 2 has been shifted to the supplementary file S1 Fig. These are archaeal OTUs, modified in the figure |
| 40 | L287 p < 0.001 is sufficient. | Agreed |
| 41 | L322 between what and buffaloes? | Modified, cattle and buffaloes |
| 42 | Figure 6 I am not able to read the text within the figure. | It is visible in original figure, but during the conversion to pdf may led to loss of resolution. Now all the figures are processed through PACE which will ensure the resolution and readability of figures |
| 43 | The discussion appears to be a simple restatement of results and comparisons to previous work. I did not find any new interpretation of the information nor new insights. | The interpretation has been added to the end of discussion section |
| 44 | There needs to be an acknowledgement and discussion of the methane yield values being lower than those reported previously. There is insufficient detail in the methods for me to suggest if the values reported here are true, or the result of errors in the technique. Sampling bias, dilution correction, gas elution, GC calibration, and emission calculation are all candidates for sources of error. | Methane values are recalculated as per Williams et al. (2019) and now the figures are comparable with the previous reports. The details for sampling, dilution, GC calibration are incorporated. |
| 45 | L343 to 345 This is in agreement with Charmley et al 2016 (doi: 10.1071/AN15365) | Added |
| 46 | L346 ‘variation’ is used when ‘difference’ is meant. | Complied |
| 47 | L356 ‘incredible’ is an emotional phrase. Please replace. | Modified |
|  | L354 to 363 This paragraph starts with microbiota and ends with methods. I don’t understand what point is being made here. | The paragraph has been removed |
| 48 | **References 27 has the author list duplicated | Corrected |
| 49 | Note, rumen is a noun used to describe one of the stomachs in bovines. Ruminal is an adjective used to describe a noun, in this case methanogens. Please use rumen and ruminal correctly throughout the manuscript. | As per suggestion, it is corrected at all the instances |
| 50 | Note the number of the verb should match the number of the subject. | Sentence corrected |
| 51 | Note, don’t use words like higher or lower etx unless you are discussing height or altitude. | Agreed and modified |
| 52 | What does this mean: Do you mean: By number, Archea constitte between 3 and 5% of the microorganisms in the rumen. Or do you mean: Archea constitute between 3 and 5% of the ruminal biomass of microorganisms. | It is the portion of archaea in the total ruminal microbiota. This has been removed from the manuscript |
| 53 | Were the animals housed in the same shed? Were they housed alongside each other? Or were they co-mingled? These issues are very important for this study. | The shed details and animal arrangement are mentioned in the methodology section |
| 54 | This equation is the equation presented by [27], but it has been shown by many researchers to be inappropriate for many reasons e.g. It does not provide units, it does not correct for molecular weights, it does not take into account background concentrations. The equation as described by Williams et al [ Williams SRO, **Moate PJ**, Hannah MC, Ribaux BE, Wales WJ, Eckard RJ. 2011. Background matters with the SF_6_ tracer method for estimating enteric methane emissions from dairy cows: A critical evaluation of the SF_6_ procedure. Animal Feed Science and Technology. 170, 265-276. doi:10.1016/j.anifeedsci.2011.08.013] is correct and has been recommended by the Global Research Alliance [Berndt, A., T. M. Boland, M. H. Deighton, J. I. Gere, C. Grainger, R.S. Hegarty, A. D. Iwaasa, J. P. Koolaard, K. R. Lassey, D. Luo, R.J. Martin, C. Martin, P. J. Moate, G. Molano, C. Pinares-Patino,  B. E. Ribaux, N. M. Swainson, G. C. Waghorn, and S. R. O.Williams. 2014. Guidelines for Use of Sulphur Hexafluoride (SF6)Tracer Technique to Measure Enteric Methane Emissions from Ruminants. Page 166. M. G. Lambert, ed. New Zealand Agric. Greenhouse Gas Res. Cent., Palmerston North, New Zealand.  Note that the methodology described by [27] is inadequate in many other respects. E.g. they only collected gas samples for between 2 and 6 hours per day. The authors should accurately and fully describe how they measured CH4 emissions, otherwise the reader may not have confidence in their results. | Agreed and modified accordingly |
| 55 | What ere the release rates (mean plus and minus SD) of SF6 from the permeation tubes? | Release rate from the permeation tube is mentioned in methane section |
| 56 | Note, mixing rations are generally defined in terms of volume. Therefore, the equation must account for molecular weights. Did you account for background concentrations of CH4 and SF6 ? What were the background concentrations of CH4 and SF6 ? Were these the same for the cattle and the buffaloes? You should report how you measured background concentrations and their values. This level of detail should match the level of detail you have used to describe microbial DNA extraction and identification. | The dilution rate and background sampling and analysis of samples are added in methodology |
| 57 | These methane yields are much smaller than usually reported for cattle fed on a forage dominant diet (e.g. Charmley et al 2016) | Methane yield is recalculated as per suggestion and now the values are comparable |
| 58 | Was the diet similar? Or was it the same except for quantity. | Yes, only intake was different between the cattle and buffaloes |
| 59 | This statement is grammatically and technically incorrect. The data presented in Table 1 shows there is indeed variation and a numerical difference in something (presumably mean concentrations). Indeed, the TVFA concentration was numerically 63% greater in cattle compared to Buffalo. Thus, you are technically incorrect in stating there was no measureable variation. You could say there was no difference in mean concentrations of either Ammonia or TVFA in ruminal fluid of cattle and Buffalo. However, you must include some measure of dispersion. I suggest you follow the journals instructions or guidelines on this. A standard error of difference or a SEM would be necessary. | The statement is modified as suggested. The greater concentration of TVFA in cattle was due to the more dry matter intake in cattle than the buffaloes (10.5 Vs 6.86). Standard error of mean has been added in the Table |
| 60 | The Table caption should explicitly state what is being presented. Mean concentrations of ammonia and total volatile fatty acids as well as molar proportions of principal volatile fatty acids in ruminal fluid of cattle and buffalo. | Caption has been modified as suggested, methane emission and methane yield also included in the table 1 and Fig. 1 has been deleted |
| 61 | I can’t see the standard errors in this Table. Please include the standard errors or some measure of dispersion. | Added |
| 62 | State cattle first throughout manuscript followed by buffaloes | Modified as suggested |
