# Supplementary material for: Comparison of enteric methane yield and diversity of ruminal methanogens in cattle and buffaloes fed on the same diet
Source: PLoS One. 2021 Aug 11;16(8):e0256048. doi: 10.1371/journal.pone.0256048 (PMC8357158; doi:10.1371/journal.pone.0256048)
Supplement: Attachment — Submitted filename: Rebuttal Letter.docx [file pone.0256048.s006.docx]

**Response to Editor and Reviewers**

We would like to thank both the reviewers for sparing their valuable time during the COVID pandemic. The comments and suggestions provided by both the reviewers are highly constructive and very much relevant ,which improved the manuscript quality substantially. This is to certify that all the suggestions have been incorporated in the revised manuscript. We are also thankful to the Editor for providing the inputs to improve the manuscript quality.

The response to the comments of reviewers and editors are appended below.

**Editor’s comment**

| S.N. | Comments/instructions | Response |
| --- | --- | --- |
| 1 | A marked-up copy of your manuscript that highlights changes made to the original version. You should upload this as a separate file labelled 'Revised Manuscript with Track Changes'. | Manuscript highlighting the changes is created separately and given the name ‘Manuscript with Track Changes |
| 2 | An unmarked version of your revised paper without tracked changes. You should upload this as a separate file labelled 'Manuscript'. | A clean manuscript is created without track changes and named as ‘Manuscript’ |
| 3 | If you would like to make changes to your financial disclosure, please include your updated statement in your cover letter. | Changes have been made in the First Revision itself |

**Reviewer # 1**

| S.N. | Comments/instructions | Response |
| --- | --- | --- |
| 1 | There is a small number of typographical errors in the manuscript, and the authors should read the manuscript carefully and remove any remaining errors. | The manuscript has been checked carefully and all the typographical errors corrected |

**Reviewer # 2**

| S.N. | Comments/instructions | Response |
| --- | --- | --- |
| 1 | Detail of the permeation tubes used is missing. How were they calibrated, and what was the mean and range of release rates? | Suggestion is incorporated. Please refer L130-L142 in the revised manuscript with track changes file |
| 2 | There is no indication of how the dilution by nitrogen was accounted for. | We have used Eqn 5.2 for the calculation of G_S_ and subsequent daily methane emission using Eqn 5.4/10.3. We appreciate the reviewer’s suggestion and felt it much more appropriate to use Eqn 8.5 for the calculation of G_S_. Accordingly the methodology has been modified (L157-167) and local elevation and atmospheric pressure have been included. However, the methane output remains same with both the Eqn 5.2 and 8.5 |
| 3 | Methane yield should be calculated as (the mean methane emission over the measurement period) divided by (the mean DMI over the measurement period). The model from Williams et al. [26] can only estimate the methane yield. You have measurements so use them instead. | Complied with, incorporated at L174-176, L279-282 & Table 1 |
| 4 | L186 replace ‘will’ with ‘were’ | Corrected |
| 5 | L200 is 13,400 rpm or g? g should be specified. | The value is specified in x g |
| 6 | L256 these are methane yield, not methane emission as specified in L255 | Agreed and modified |
| 7 | 274 OTUs – check journal requirements. Many journals use the singular abbreviation to also mean the plural. This would mean OTU is used here. | Checked with Journal articles, both the terminology have been used. However, as per instructions, we have changed OTUs with OTU |
| 8 | Figure 4 The text in figure 4 is unreadable in the copy provided. | Fig 4 is modified with better resolution |
| 9 | L424 which country? | India, modify accordingly (L457) |
| 10 | ‘Distribution’ is used when discussing the proportion of the archaea population that is occupied by a particular genera or species. I think ‘proportion’ should be used in these cases. | Necessary changes have been made |
| 11 | if the dilution correction is an explanation for the low calculated methane yields (mean 13.4 g/kg DMI). | If we checked the dilution with both the Eqn 5.2 and Eqn 8.5 and found similar DP. The methane yield is within the acceptable range of 12-30 g/kg DMI. However, the values are lower than that reported by Charmley et al. The reason for the lower methane emission/yield could be the due to the methane inhibitory substances in the diet. The Napier grass constituted 70% of the diet and known to contains tannins and saponins, which inhibit methane emission. Please refer L398-406  To confirm the presence of tannins/saponins in the Napier grass, we also did the qualitative analysis and found the tannins presence in Napier grass (qualitative test photographs are given below for your kind reference) |

**Qualitative analysis**

Tannins (+)/Saponins (-)

Fig 4
