# Supplementary material for: Comparison of enteric methane yield and diversity of ruminal methanogens in cattle and buffaloes fed on the same diet
Source: PLoS One. 2021 Aug 11;16(8):e0256048. doi: 10.1371/journal.pone.0256048 (PMC8357158; doi:10.1371/journal.pone.0256048)
Supplement: Attachment — Submitted filename: Rebuttal Letter.docx [file pone.0256048.s007.docx]

**Response to Editor and Reviewers**

We would like to thank the reviewer for the prompt evaluation and providing the critical inputs, which certainly improved the manuscript. This is to certify that all the suggestions have been incorporated in the revised manuscript. We are also thankful to the Editor for providing the inputs to improve the manuscript quality. The response to the comments of reviewers and editors are appended below.

**Editor’s comment**

| S.N. | Comments/instructions | Response |
| --- | --- | --- |
| 1 | A marked-up copy of your manuscript that highlights changes made to the original version. You should upload this as a separate file labelled 'Revised Manuscript with Track Changes'. | Manuscript highlighting the changes is created separately and given the name ‘Manuscript with Track Changes |
| 2 | An unmarked version of your revised paper without tracked changes. You should upload this as a separate file labelled 'Manuscript'. | A clean manuscript is created without track changes and named as ‘Manuscript’ |
| 3 | Please review your reference list to ensure that it is complete and correct. If you have cited papers that have been retracted, please include the rationale for doing so in the manuscript text, or remove these references and replace them with relevant current references. Any changes to the reference list should be mentioned in the rebuttal letter that accompanies your revised manuscript. | Reference list has been checked for retracted paper if any. We did not find any retracted paper in our list.  As per the reviewer’s suggestion, the correct reference for SN 25 has been replaced |

**Reviewer # 2**

| S.N. | Comments/instructions | Response |
| --- | --- | --- |
| 1 | I suggest a minor adjustment at line 156. Replace 'The concentrations of methane...' with 'The physical dilution with nitrogen was mathematically accounted for using the equation of Lassey et al. with adjustment for local elevation and atmospheric pressure.' | The sentence has been changed as suggested (L156-158 in the marked manuscript) |
| 2 | Please note that the reference at line 158 is to the wrong chapter of the guidelines. The correct reference should be Lassey KR, Martin RJ, Williams SRO, Berndt A, Iwassa AD, Hegarty RS, et al. Analyses of breath samples. In: Lambert MG, editor. Guidelines for use of sulphur hexafluoride (SF6) tracer technique to measure enteric methane emissions from ruminants. New Zealand: New Zealand Agricultural Greenhouse Gas Research Centre; 2014. p. 166. | Thank you very much, the correct reference at same SN (25) has been incorporated in place of the earlier one. |
